# A wrinkle in timers: evolutionary rewiring of conserved biological timekeepers

**DOI:** 10.1016/j.tibs.2025.01.006

**Published:** 2025-02-13

**Authors:** Rebecca K. Spangler, Keya Jonnalagadda, Jordan D. Ward, Carrie L. Partch

**Affiliations:** 1 Department of Chemistry and Biochemistry, University of California – Santa Cruz, Santa Cruz, CA 95064, USA; 2 Department of Molecular, Cell, and Developmental Biology, University of California – Santa Cruz, Santa Cruz, CA 95064, USA; 3 Center for Circadian Biology, University of California – Santa Diego, La Jolla, CA 92093, USA; 4 Howard Hughes Medical Institute, University of California – Santa Cruz, Santa Cruz, CA 95064, USA

## Abstract

Biological timing mechanisms are intrinsic to all organisms, orchestrating the temporal coordination of biological events through complex genetic networks. Circadian rhythms and developmental timers utilize distinct timekeeping mechanisms. This review summarizes the molecular basis for circadian rhythms in mammals and *Drosophila*, and recent work leveraging these clocks to understand temporal regulation in *Caenorhabditis elegans* development. We describe the evolutionary connections between distinct timing mechanisms and discuss recent insights into the rewiring of core clock components in development. By integrating findings from circadian and developmental studies with biochemical and structural analyses of conserved components, we aim to illuminate the molecular basis of nematode timing mechanisms and highlight broader insights into biological timing across species.

## Timekeeping in nature

Molecular timing mechanisms are vital in orchestrating life processes, directing events from cellular functions to organismal behaviors to optimize fitness and fecundity. Biological timers are tightly regulated and often integrate external signals to align internals processes with the outside world. They operate across diverse timescales, from the multi-year cycles of cicadas [1] to the seconds-long rhythms of cardiac pacemakers [2]. At the cellular level, timers are typically generated through genetic programs broadly categorized as either hourglass timers or self-sustaining oscillators (Box 1) [3].

While some biological timers are relatively simple, like the circadian oscillator of the cyanobacterium *Synechococcus elongatus* that requires only three proteins to generate ~24-h cycles [4], many temporal mechanisms are more complex, often integrating elements of both hourglass and oscillatory timers. For example, the repeated formation of somites in vertebrate segmentation clocks follows an hourglass pattern that is driven by an underlying oscillatory timer [5]. Similarly, in *C. elegans* development, the interdependence of an hourglass and oscillatory timer ensures proper embryonic development, with both timers being essential for successful maturation to adulthood [6].

Biological timers regulate a variety of processes across different timescales. **Circadian rhythms** (see Glossary) coordinate processes like sleep–wake cycles and body temperature fluctuations. Ultradian (<24-h) rhythms control processes like cell cycling and rapid eye movement (REM) sleep cycles. Infradian (>24-h) rhythms govern patterns like menstrual cycles and migration patterns [3]. Each temporal process functions through distinct molecular mechanisms to establish correct timescales. Despite advances in the study of these timekeeping processes individually, there remain significant gaps in our understanding of their evolution and functional repurpose.

The model organism, *C. elegans*, is a particularly valuable system for novel investigation into the evolution and adaptation of biological timers. Several proteins essential for mammalian and *Drosophila* circadian rhythms play dual roles in *C. elegans*, controlling both ~24-h oscillatory gene expression patterns in adults and developmental timing of larvae. This highlights how circadian gene orthologs can be repurposed to regulate distinct biological timing mechanisms. [7–14]. The following review brings together recent work from the metazoan circadian and *C. elegans* developmental fields to discuss biological timing mechanisms across evolution and timescales from a biochemical perspective. We highlight recent progress in each field, focusing on connections between timekeeping processes and how they have diverged or been rewired through evolution.

## Molecular basis for mammalian circadian rhythms

**Figure 1.**
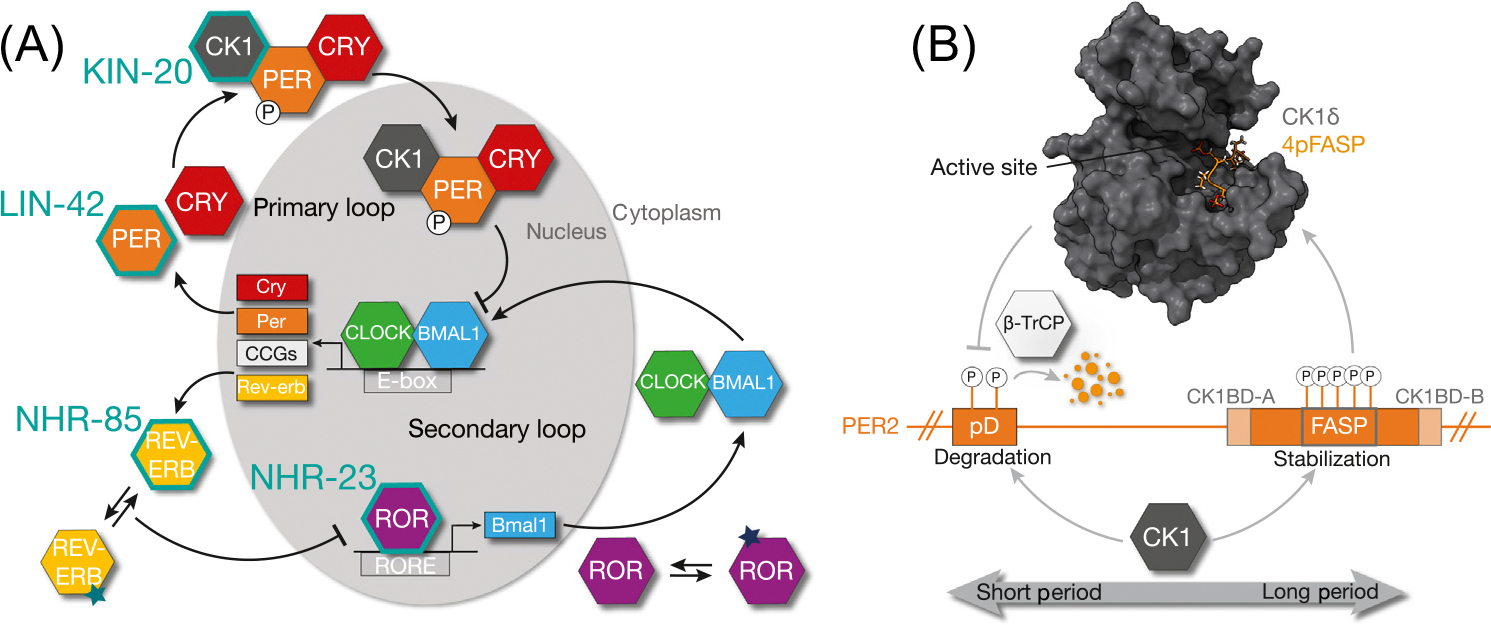
Mammalian circadian rhythms. (A) Cartoon schematic depicting the interlocked set of transcription–translation feedback loops (TTFLs) that govern mammalian circadian rhythms. *Caenorhabditis elegans* homologs are indicated in teal (KIN-20/CK1, LIN-42/PER, NHR-85/REV-ERB, and NHR-23/ROR). Stars on mammalian nuclear hormone receptors (NHRs), REV-ERB and ROR, are representative of ligand binding. (B) Schematic illustrating the mammalian phosphoswitch that dictates CK1-dependent regulation of PER stability. Crystal structure of human CK1δ (blue) bound to phosphorylated PER2 FASP (orange, 4pFASP) peptide, PDB: 8d7o. Abbreviations: CCG, clock-controlled gene; FASP, familial advanced sleep phase; pD, phosphodegron.

As a self-sustaining oscillator, circadian rhythms are one of the most well-studied timing mechanisms at the molecular, cellular, and organismal levels. Daily light and temperature cycles are anticipated by organisms and manifest as a wide range of biological processes to keep internal clocks aligned with the external environment [15]. Eukaryotic ~24-h rhythms are generated at the molecular level by an interlocked set of **transcription–translation feedback loops (TTFLs)** that result in the cyclic expression of clock-controlled genes (CCGs) including certain core clock components (Figure 1A) [16]. These TTFLs regulate ~40% of mammalian protein coding genes, giving rise to a single peak of expression once per day [17]. The primary loop establishes the period of circadian rhythms. It is governed by the heterodimeric transcription factor CLOCK:BMAL1 [circadian locomotor output cycles protein kaput (CLOCK); brain and muscle ARNT-like 1 (BMAL1), or ARNTL], members of the bHLH-PAS [basic helix–loop–helix (bHLH); Period-Arnt-Sim, (PAS)] transcription factor family. This complex activates the transcription of over a thousand CCGs [18], including its own negative regulators, PERIOD (three isoforms; PER1, PER2, and PER3) and CRYPTOCHROME (two isoforms; CRY1 and CRY2). In general, PERs and CRYs accumulate and interact in the cytoplasm, recruit a crucial circadian kinase, Casein Kinase 1 (three isoforms; CK1δ1, CK1δ2, and CK1ε; hereafter referred to as CK1), and translocate into the nucleus as large complexes to repress CLOCK:BMAL1 activity (Figure 1A) [19].

CK1 has a critical, post-translational role in setting the ~24-h period by phosphorylating PER1 and PER2 to regulate their abundance, with PER2 being the better-studied protein [20,21]. Two motifs within the CK1-binding domain (CK1BD; CK1BD-A and CK1BD-B) stably anchor PER2 to CK1 throughout the day [22] allowing a **phosphoswitch** mechanism to control PER2 stability and introduce a phase-specific delay in its degradation necessary for correct circadian period (Figure 1B) [20,21,23]. Phosphorylation of a degron [phosphodegron (pD)] downstream of the PAS-B domain recruits the E3 ubiquitin ligase, ß-TrCP, marking PER2 for degradation. CK1 activity at the familial advanced sleep phase (FASP) region – a five-serine cluster embedded within the CK1BD-A and CK1BD-B – inhibits activity at the pD (Figure 1B) [20,23–26]. Stable CK1-PER anchoring enables phosphorylation of weak, non-consensus sites [20,22] and phosphorylation of the five serines in the FASP is rate-limited by the initial non-consensus serine, which primes downstream consensus residues following the pSXX**S** consensus motif (with phosphorylated serine indicated in bold) [27]. FASP phosphorylation inhibits CK1 through docking into the substrate-binding cleft near the CK1 active site (Figure 1B). Disrupting phosphorylation within the FASP region shortens the circadian period in mammalian cells [25]. Not only has CK1 been linked to control of temperature compensation and thus maintains stable periods at biologically relevant temperatures [26,28], CK1 also participates in the removal of CLOCK:BMAL1 from DNA via PER- and CRY-dependent phosphorylation of CLOCK, further establishing a central role for CK1 in circadian rhythms beyond its influence on PER [29].

The secondary TTFL is governed by two antagonistic **nuclear hormone receptors (NHRs),** the retinoic acid receptor-related receptors (RORα, RORß, and RORγ) and reverse-ERB (REV-ERBα and REV-ERBß). This loop influences the stability and amplitude of circadian rhythms by transcriptionally activating (RORs) or repressing (REV-ERBs) the expression of CLOCK, BMAL1, and the CLOCK paralog, neuronal PAS-domain protein 2 (NPAS2) (Figure 1A) [30]. All three ROR proteins have been implicated in circadian rhythms; however, RORß is mainly expressed in the retina and pineal gland, while RORα/γ have a broader range of expression and additional roles in metabolism, embryonic development, and immune functions [31]. The REV-ERB isoforms are largely functionally redundant and display similar expression patterns across cell types [30]. Both RORα/ß/γ and REV-ERBα/ß are ligand-regulated and thought to link circadian rhythms with metabolism along with another NHR, PPARγ [32–35]. In addition to synthetic derivatives, ROR proteins bind to cholesterol derivatives while REV-ERB binds porphyrins [32]. Crosstalk between the primary and secondary loops is evident through additional mechanisms of PER regulation, specifically of NHRs, highlighting the intricate network of interlocked TTFL regulation [18,35,36].

## Molecular basis for *Drosophila* circadian rhythms

**Figure 2.**
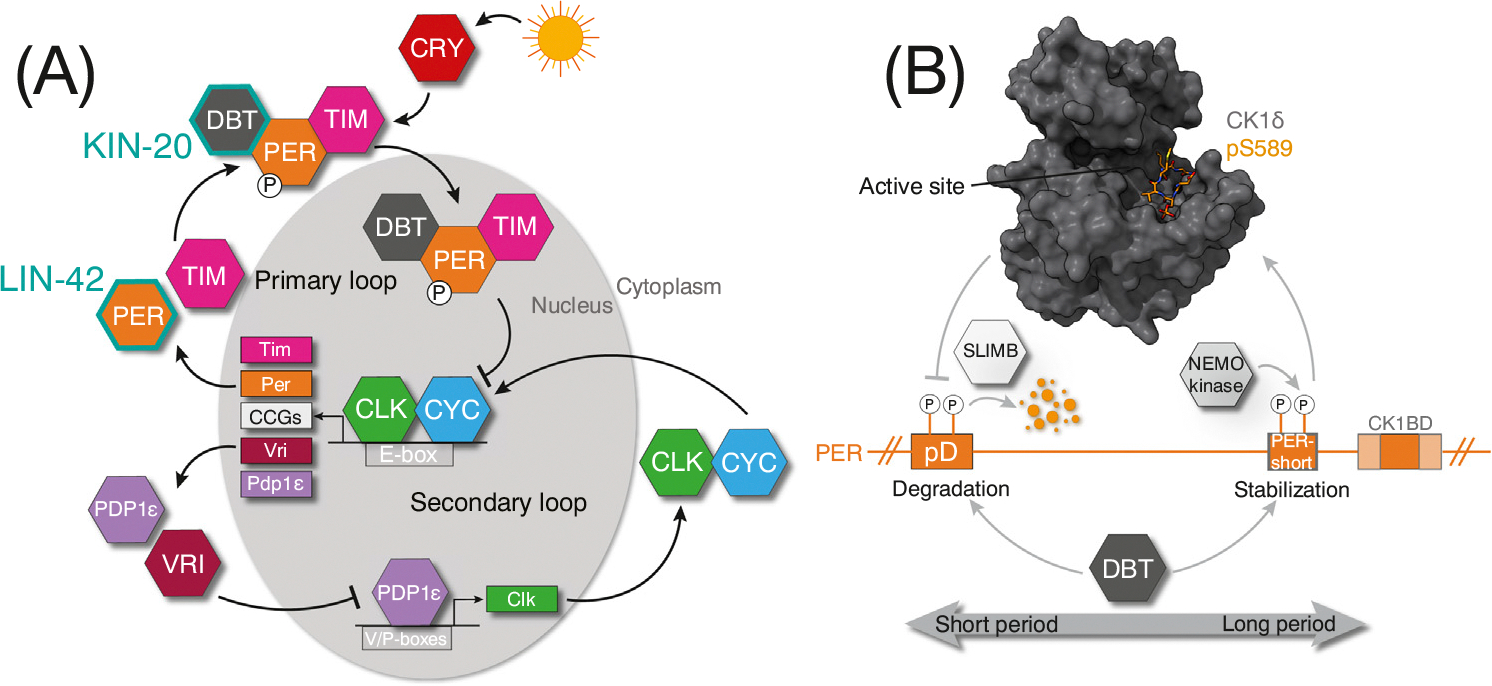
*Drosophila* circadian rhythms. (A) Cartoon schematic depicting the interlocked set of transcription–translation feedback loops (TTFLs) that govern *Drosophila* circadian rhythms. *Caenorhabditis elegans* homologs are indicated in teal (KIN-20/DBT, LIN-42/PER). (B) Schematic illustrating the *Drosophila* phosphoswitch that dictates NEMO kinase and DBT-dependent regulation of PER stability. Crystal structure of human CK1δ (blue) bound to *Drosophila* phosphorylated S589 (orange, pS589) PER-short peptide (orange, 4pFASP), PDB: 8d7p. pD, phosphodegron. Abbreviation: CCG, clock-controlled gene.

Like mammals, *Drosophila* circadian rhythms are generated via interlocked TTFLs; however, the core components differ in several regards while maintaining a similar molecular architecture (Figure 2A). In the *Drosophila* primary loop, like mammalian CLOCK:BMAL1, CLOCK and CYCLE are bHLH-PAS transcription factor subunits that form a heterodimer and rhythmically bind to E-box elements [37]. A single PER isoform is conserved in *Drosophila*; however, another repressor, Timeless (TIM), replaces mammalian CRY in the core loop (Figure 2A) [37,38]. While the role of CLOCK:CYCLE as positive elements in the primary loop is conserved, CLOCK is the main driver of transcriptional activation since CYCLE lacks a transactivation domain [39]. In mammals, the BMAL1 transactivation domain is required for CLOCK:BMAL1 activity in cells and *in vivo* [40], and this domain is sequestered from coactivators by CRY to directly inhibit CLOCK:BMAL1 [41]. This evolutionary divergence is likely the reason that *Drosophila* CRY is not a transcriptional repressor and instead has been coopted to an auxiliary role as a photoreceptor, directly sensing light and passing the information to the core loop via control of TIM stability (Figure 2A) [37].

Like mammalian CK1, the *Drosophila* homolog, Doubletime [DBT, also known as Discs Overgrown (DCO)], stably anchors to PER and ultimately regulates circadian period through a functionally similar phosphoswitch (Figure 2B) [42–46]. In contrast to CK1, which both primes and phosphorylates consensus serines in PER, *Drosophila* utilizes the NEMO kinase to prime PER substrates for DBT [47]. The *Drosophila* PER-short domain functions like the mammalian FASP region to increase PER stability by reducing kinase activity at an upstream phosphodegron recognized by *Drosophila* ß-TrCP (SLIMB) [43]. While still proximally close, PER-Short is located upstream to the CK1BD yet exhibits similar phosphorylation-dependent attenuation of CK1 activity [25,46,48]. Differing modes of PER-Short and FASP peptide binding along the kinase active site demonstrate that different mechanisms of recognition can be used to bind and inhibit CK1 (Figure 2B) [25]. Notably, DBT stably associates with Bride of Doubletime (BDBT), a noncanonical FK506-binding protein. BDBT influences DBT activity on *Drosophila* PER, ultimately affecting circadian period and highlighting an additional means of DBT regulation absent in the mammalian system [49,50].

The secondary loop of the *Drosophila* TTFLs is controlled via PAR domain protein 1ε (PDP1ε) and Vrille (VRI), which activate and repress CLOCK expression, respectively (Figure 2A) [37,51]. Unlike RORα/ß/γ and REV-ERBα/ß that regulate the mammalian secondary TTFL, *Drosophila* orthologs, *Drosophila* hormone receptor 3 (DHR3, also known as HR46) and ecdysone-induced protein 75B (E75), respectively, regulate a linear timer in metamorphosis. Both DHR3 and E75 are NHRs induced by ecdysone initiating a cascade of gene expression necessary for larval molting and metamorphosis. In contrast to their mammalian counterparts that compete for DNA binding, E75 forms a heterodimer with DHR3, repressing its transcriptional activity. This repression is relieved when E75 is removed, allowing DHR3 to activate downstream genes [52,53]. Although DHR3 is considered an **orphan receptor**, its role in ecdysteroid-driven metamorphosis suggests it may also bind steroid hormones, like its mammalian counterpart ROR α/ß/γ.

## Biological timing mechanisms in *C. elegans*

The genetic and cellular tractability of *C. elegans* make them especially suited for developmental and chronobiological studies [54]. However, the molecular basis of biological timekeeping in this organism remains unclear, despite the conservation of multiple metazoan circadian clock components. While mammalian PER (*d*PER), CK1 (*d*DBT), ROR (*d*HR3), and REV-ERB (*d*E75) have *C. elegans* counterparts in LIN-42, KIN-20, NHR-23, and NHR-85, respectively, notably absent are clear homologs for the bHLH-PAS transcription factor subunits, CLOCK (*d*CLOCK), BMAL1 (*d*CYCLE), and CRY, the mammalian primary loop repressor (Figures 1A and 2A). Little is known about the role of *C. elegans* TIM-1, ortholog to *Drosophila* TIM, in nematode development [55]. Given its prominent role in DNA replication and its conserved interaction with Tim Interacting Protein (TIPIN-1) in mammals, *C. elegans* TIM-1 may function more similarly to mammalian than *Drosophila* TIM [56]. Despite the absence of canonical TTFL activating arm components, oscillatory processes in *C. elegans* might arise from alternative regulatory mechanisms. For example, *C. elegans* components could function in a simpler, negative feedback loop decoupled from bHLH-PAS-driven transcriptional activation. This aligns with findings in noncanonical timekeeping systems, such as cyanobacteria, where oscillatory dynamics are driven by post-translational modifications rather than transcriptional feedback [4,57].

Conserved circadian orthologs also serve non-circadian roles. For example, *C. elegans* NHR-23 is an essential regulator of spermatogenesis, and its expression does not oscillate in the spermatogenic germline [58]. Similarly, *Drosophila* PER and TIM are constitutively expressed in fly ovaries. These examples underscore how clock components can be repurposed for distinct biological processes beyond circadian timekeeping [59–61].

### The circadian rhythms of *C. elegans*

Over a decade ago, ~24-h rhythms were identified in adult *C. elegans* behaviors, including loco-motion, olfactory responses, and some metabolic functions, although these patterns are less robust than other model organisms [54]. A bioluminescent assay confirmed ~24-h gene expression oscillations in individual and populations of animal. However, these rhythms likely reflect an hourglass timer rather than a self-sustaining oscillator, as they dampen quickly under constant conditions yet entrain robustly to cycles in light and temperature [7]. The lack of sustained rhythmicity beyond the first day in constant conditions means they do not meet the classical definition of circadian rhythms, which require self-sustained oscillations under constant conditions. Instead, these ~24-h rhythms may rely on non-TTFL mechanisms and/or environmental cues to maintain temporal organization.

While it remains unclear whether these ~24-h rhythms serve a specific biological function, LIN-42 and KIN-20, homologs to PER and CK1/DBT, are implicated in its regulation [7,8]. Recent transcriptome analyses also identified NHR-23, the ROR/DHR3 ortholog, as essential for adult gene expression patterns [9], demonstrating the conserved timekeeping function of these orthologs. These findings highlight the importance of *C. elegans* chronobiological studies, as the molecular mechanisms behind these orthologs’ ability to drive two distinct oscillators and count multiple periods within a single organism are unknown. How these timers transition from developmental to adult circadian roles remains a key question.

### Hourglass and oscillatory timers govern *C. elegans* development

From embryo to reproductive adulthood, *C. elegans* develop through four larval stages [62]. Two independent but interconnected biological timers drive progression through this development. The **heterochronic pathway** is an hourglass timer that controls the serial progression of stage-specific cellular events (see the bottom of Figure I in Box 2) [62]. An oscillatory **molting** timer controls the tempo of development and coordinates apical extracellular matrix regeneration that allows for organism growth (see the top of Figure I in Box 2) [63,64]. While the molting cycle shares some features with hourglass mechanisms – such as accumulation of certain factors over time – the periodicity and coordination with larval stage transitions are consistent with an underlying oscillatory network. In contrast to the circadian periods of metazoans, the genetic oscillator that governs nematode molting peaks once per larval stage [65]. The periodicity of molt cycles is tightly regulated, occurring at regular 8–10-h intervals that are precisely coordinated with heterochronic, developmental progression [64,66] (Box 2). Like circadian rhythms, molting is driven by oscillatory gene expression patterns, with over 20% of the larval transcriptome (~3700 genes) exhibiting rhythmic gene expression synchronized with larval stages. Unlike circadian rhythms, nematode molting operates on a shorter period, is not temperature compensated, and has a finite number of four oscillations [65]. While the involvement of circadian rhythm orthologs in *C. elegans* developmental timing has been recognized for decades, until recently, very little was known about the underlying biochemical mechanisms that drive this distinct biological timer [12–14,55,67–70].

## Biochemical insights into *C. elegans* temporal development

### KIN-20

Among *C. elegans* development proteins with orthologs that regulate metazoan circadian rhythms, KIN-20 is the most conserved, sharing nearly 80% sequence identity to human CK1 within its kinase domain. The active sites for KIN-20, CK1, and DBT are 100% identical, underscoring the remarkable conservation seen in this family of serine/threonine kinases [26,71]. Despite essential roles in processes like Wnt signaling, cell division, apoptosis, and circadian rhythms [72,73], the molecular mechanisms regulating CK1 family activity remain largely unknown, particularly regarding KIN-20’s role in *C. elegans* development. Beyond its role in circadian rhythms in adult *C. elegans*, KIN-20 is essential for axon branching during nervous system maturation – a function that has been minimally explored in mammals, with a single study identifying CK1 activity as necessary for neurite outgrowth in cultured cell models. Additionally, like LIN-42, KIN-20 also regulates the expression of heterochronic pathway components and an additional role in the molting cycle is demonstrated by *kin-20* mutations that result in irregularly timed molting during development [11,55,71,74].

**Figure 3.**
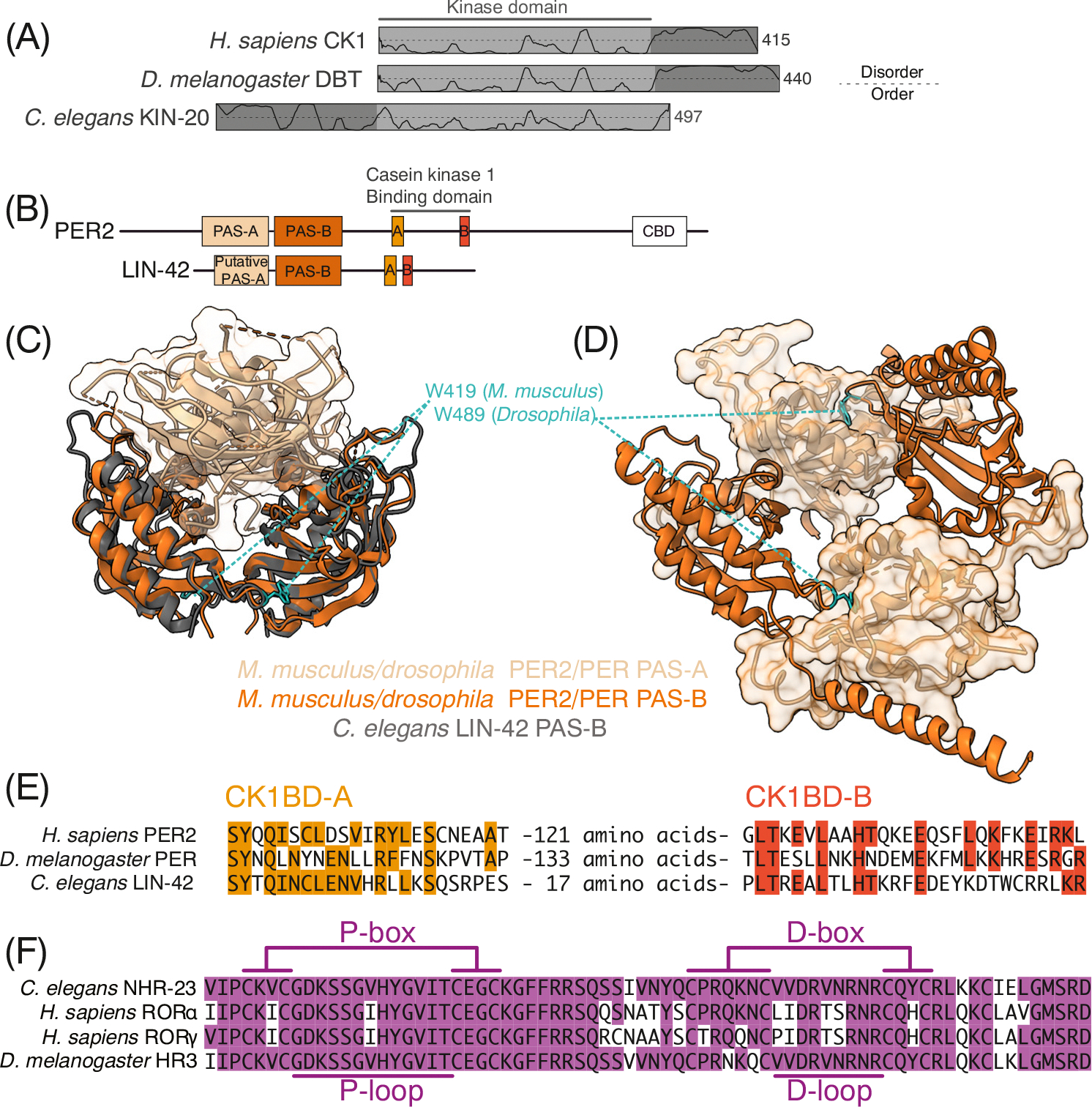
Biochemical insights into *Caenorhabditis elegans* timekeepers. (A) Domain architecture of *C. elegans* KIN-20, *Homo sapiens* CK1, and *Drosophila* DBT. Traces indicate the propensity for intrinsic disorder. (B) Functional domain architecture of LIN-42 compared with mammalian PER2 and *Drosophila* PER. Structured domains are indicated as boxes. PAS, PER-ARNT-SIM; CK1BD, casein kinase 1-binding domain; CBD, CRY-binding domain. (C) LIN-42 PAS-B dimer (gray, PDB: 8gci) aligned to mouse PER2 tandem PAS structure (orange, PDB: 3gdi, PAS-B cartoon representation, PAS-A surface representation). (D) *Drosophila* PER tandem PAS structure (orange, PAD: 1wa9, PAS-B cartoon representation, PAS-A surface representation). (E) Sequence alignment of the CK1BD motifs of human PER2, *Drosophila* PER, and LIN-42. The asterisk (*) indicates conserved residue that mediates PER2:CK1 interaction [22]. (F) Sequence alignment of the DNA-binding domains of *C. elegans* NHR-23, *H. sapiens* RORα, *H. sapiens* RORγ, and *Drosophila melanogaster* HR3. P- and D-box/loop regions utilized for DNA interaction and dimerization respectively, are indicated.

Difficulties in purifying recombinant KIN-20 have limited biochemical insights, leading most studies to rely on genetic approaches. Recent work has leveraged the extreme conservation between KIN-20 and its mammalian homologs to investigate potential *C. elegans* substrates [71,74]. This kinase domain-based approach is limited by the extended sequence found on the N terminus of KIN-20’s main isoform. While CK1 and DBT possess autoinhibitory C-terminal tails of ~100 residues [75,76], KIN-20 lacks this C-terminal extension. Instead, its primary isoform contains 183 N-terminal residues that are predicted to be disordered (Figure 3A). Whether the N terminus of KIN-20 functions similarly to the autoinhibitory C termini of CK1 and DBT remains unknown. Future work in this area will not only enhance our understanding of the molecular basis of circadian rhythms and *C. elegans* development but will also shed light on the broader regulation of CK1 family kinases.

### LIN-42

LIN-42, the *C. elegans* PER ortholog, is a transcriptional repressor that controls heterochronic microRNA patterning and is likely involved in crosstalk between the heterochronic pathway and molting [12,14,68–70,77,78]. Unlike PER, whose mechanistic functions are well understood, little is known about the molecular basis for LIN-42 function. Like the mammalian and *Drosophila* PER proteins, LIN-42 is predicted to be mainly disordered. However, multiple structured domains critical for PER functions are conserved, including tandem PAS-domain architecture and two helical motifs involved in CK1/DBT-binding, suggesting that some functions of PER may be conserved in LIN-42 (Figure 3B) [13,14,79].

In mammals, PER proteins contain N-terminal tandem PER-ARNT-SIM (PAS-A and PAS-B) domains that mediate homodimerization [79–82]. While the functional role of PER homodimerization is conserved in mammals and *Drosophila*, the mechanistic strategies utilized differ significantly between the two [80,83]. In mammals, PER dimerization occurs through antiparallel PAS-B interfaces with a conserved tryptophan (W419 in mice) playing a crucial role (Figure 3C) [80]. Conversely, *Drosophila* PER dimerization is stabilized by PAS-A interactions with a conserved tryptophan (*Drosophila* W482) in a loop of the PAS-B domain and a helix within the same domain, known as αF (Figure 3D) [84]. Although the complete structure of the LIN-42 N terminus remains unresolved, we recently determined that the PAS-B domain utilizes a dimerization strategy similar to human/mouse PER2, demonstrating that *C. elegans* interactions align more with mammals than with *Drosophila* (Figure 3C,D) [8]. Still, the unresolved structure of the putative PAS-A region indicates that functional differences likely exist. Supporting this notion, our recent precise deletion of the *lin-42* sequence encoding for putative PAS-A and PAS-B domains using CRISPR, as described in preprint [71], resulted in only mild heterochronic phenotypes and an overall molting pattern resembling that of wild type animals. This suggests that although the LIN-42 PAS region, while evolutionarily related to mammalian PER PAS domains, may have distinct functional roles in *C. elegans*.

LIN-42 also contains a conserved CK1-binding domain (CK1BD), which has two binding motifs: CK1BD-A and -B [79]. These motifs show modest conservation across humans, *Drosophila*, and *C. elegans*, notably retaining a leucine residue that, when mutated in mammals, abolishes stable PER2-CK1 association (Figure 3E) [22,24]. Although previous work suggested that *lin-42* and *kin-20* act in separate genetic pathways [11], we recently demonstrated in a preprint [71] that LIN-42 retains enough conservation within its CK1BD to mediate binding to CK1 *in vitro*, and that LIN-42 interacts with KIN-20 *in vivo*. The LIN-42 CK1BD, KIN-20, and KIN-20 activity are required for proper molting timing. While we demonstrated that LIN-42 exhibits a conserved mode of feedback inhibition on CK1, it remains unclear whether this is utilized in a phosphoswitch mechanism to regulate LIN-42 abundance like the mammalian and D*rosophila* systems [71].

As central pacemakers of development and circadian rhythms, LIN-42 and PER offer key evolutionary insights into both fields of chronobiology. Studying LIN-42 not only has the potential to deepen our understanding of PER function by highlighting conserved features, but it may also uncover novel mechanisms unique to *C. elegans* development, providing insight into two distinct biological oscillators.

### Nuclear hormone receptors

The *C. elegans* genome encodes 283 NHRs – significantly more than the 48, 49, or 21 found in humans, mice, and *Drosophila*, respectively [85,86]. Among these, NHR-23 and NHR-85, homologous to mammalian ROR (DHR3) and REV-ERB (*d*E75), are considered orphan receptors. NHR-23 is essential for *C. elegans* molting, embryonic development, and spermatogenesis [9,10,58,67,87–90]. By contrast, NHR-85 has a more limited role, mildly affecting brood size and molt into dauer, a specialized stress-resistant larval stage [91–93]. Given the roles of DHR3 and E75 in *Drosophila* molting, NHR-23/85 may use similar mechanisms to regulate *C. elegans* development. However, recent findings show NHR-23 is also critical for driving ~24-h rhythmic gene expression in adults under specific conditions [9], raising questions about its precise molecular mechanisms and whether they align more with its mammalian or *Drosophila* orthologs.

NHR-23 and NHR-85 are ~25–40% sequence identical to their mammalian and *Drosophila* counterparts, with ≥60% identity in their DNA-binding domains (DBDs). At 81%, the NHR-23 DBD shows particularly high similarity to the DBD of DHR3 (Table 1). Structurally, NHR DBDs typically contain two zinc finger motifs: the P-box, which confers DNA-binding specificity, and the D-box, involved in dimerization [94]. NHR-23, DHR3, and ROR all have similar P-boxes and all bind ROR-response elements (ROREs) (Figure 3F) [34,93]. However, the NHR-23 D-box shows more divergence from RORs compared with DHR3, suggesting a conserved mode of dimerization between NHR-23/DHR3 and NHR-85/E75 (Figure 3F).

Recent work demonstrated NHR-23/NHR-85 RORE-binding cooperativity and transcriptional activation of a conserved heterochronic microRNA [93], a mechanism distinct from the antagonistic roles of mammalian ROR/REV-ERB. While heterodimerization is conserved with *Drosophila* DHR3/E75, the cooperativity is unique to *C. elegans* [53,95]. The same study also discovered LIN-42 interactions with 65 NHRs, including NHR-85 but not NHR-23, and several NHRs whose mammalian orthologs also bind PER2 [93]. This suggests parallels between LIN-42/NHR crosstalk in *C. elegans* and PER2 regulation within the mammalian secondary loop [35,36].

Additional studies have also revealed a direct regulatory feedback loop between NHR-23 and *let-7*, a crucial heterochronic microRNA that specifies larval seam cell fates [88]. This feedback interaction not only demonstrates a link between the heterochronic pathway and molting cycle, but also further crosstalk of LIN-42 and NHRs. LIN-42 and *let-7* mutually inhibit one another [68–70,78,96], and the presence of complementary *let-7*-binding sites, as well as ROREs in the *lin-42* promoter, suggest LIN-42 and NHR-23 may contribute to a self-sustaining molecular oscillator [88,93] although the full details of such a regulatory network remain elusive.

The degree of conservation in the NHR-23 ligand-binding domain (LBD) is less pronounced compared with its DBD, with this region less than 30% identical to mammalian RORs and DHR3 (Table 1). However, given that NHR-23 is implicated in diet-induced acceleration of development as well as dietary restriction-induced longevity [97,98], a major question in the field is whether the ligand-binding properties of ROR are conserved in NHR-23 to control *C. elegans* development. Future research is needed to explore the ligand-binding properties of NHR-23 and NHR-85 to identify if the regulatory mechanisms align with those of their mammalian counterparts, potentially revealing new insights into conserved and divergent functions across species.

## Concluding remarks

While much remains to be uncovered, integrating insights from circadian biology with biochemical and structural analysis is beginning to shed light on the molecular mechanisms underlying nematode developmental timing. A key question in this area is how conserved components like KIN-20, LIN-42, and NHR-23 shift from ~8–10-h developmental cycles to ~24-h rhythms in adulthood and whether alignment with daily environmental cycles is necessary during larval stages. Studying the influence of environmental cues on nematode development could uncover ancient regulatory mechanisms, including the role of LBDs in nuclear receptors like NHR-23, which may act as environmental sensors with conserved timing functions. Additionally, understanding if the molting genetic oscillator operates via a simplified or hybrid mammalian/*Drosophila* TTFL may reveal evolutionary adaptations in developmental timing. Together, these investigations will not only deepen our knowledge of developmental and circadian timing, but also highlight the evolutionary adaptability of biological timing mechanisms across species (see Outstanding questions).

## Figures and Tables

**Table 1.** Percent identity matrix of NHR-23 and NHR-85 to their mammalian and *Drosophila* homologs

Percent identity to NHR-23	Full length (%^a^)	DBD (%)	LBD (%)
*Homo sapiens* RORα	30	65	21
*Homo sapiens* RORγ	32	62	23
*Drosophila melanogaster* HR3	39	81	29
Percent identity to NHR-85			
*Homo sapiens* REV-ERBα	32	65	22
*Homo sapiens* REV-ERBβ	33	65	20
*Drosophila melanogaster* E75	27	59	15

Abbreviations: DBD, DNA-binding domain; LBD, ligand-binding domain.

a Percentages were generated using Clustal Omega multiple sequence alignment tool (EMBL) [99].

## Box 1. Defining hourglass and oscillatory timers

Typically, chronobiological mechanisms are categorized as one of the following.

### Hourglass timers

These timers are prevalent in unidirectional processes, primarily development and aging, where a specific sequence of events is required. These timers measure temporal durations or periods by relying on rate-determining steps, often involving the accumulation or degradation of critical factors that trigger sequential events. Hourglass mechanisms are nonredundant, often utilizing multiple thresholds to orchestrate a cascade of events, ensuring that biological processes proceed within precise timescales and in the correct order.

### Oscillatory timers

These timers generate repetitive patterns and are typically governed by feedback loops, both positive and negative, that generate self-sustaining rhythms. Unlike hourglass timers, which gauge time duration, oscillatory timers determine time points at consistent intervals. Self-sustaining oscillators, such as the cell cycle, neural oscillations, and ovarian and ultradian cycles, synchronize with biological timescales but are also prevalent in processes that keep time with environmental signals, such as circatidal, circalunar, circannual, and circadian rhythms.

## Box 2. *Caenorhabditis elegans* development is coordinated via two biological timing mechanisms

Each *C. elegans* molting cycle is defined as having a feeding, lethargus, and ecdysis phase. Lethargus is a sleep-like state where the animals do not move or eat, and ecdysis describes the process where the animals wriggle out of their old cuticle. The pink panel (Figure I) represents the linear, heterochronic pathway that governs stage-specific differentiation of stem cell-like cells (seam cells). The lineage of representative seam cells (H0, H1, and V1-V4) is depicted. The light-gray circles represent anterior daughters that fuse and the gray diamonds represent stem seam cells.

## Outstanding questions

How do *C. elegans* KIN-20, LIN-42, and NHR-23 transition from an ~8–10-h developmental timer to a ~24-h circadian cycle in adulthood?

Do larval stage animals require alignment with ~24-h environmental cycles?

What molecular mechanisms drive the *C. elegans* molting gene regulatory network, and could it involve a simplified TTFL? Does this network resemble mammalian or *Drosophila* circadian TTFLs or represent a unique hybrid?

Does *C. elegans* use the ligand-binding domains of NHR-23 or other NHRs to sense environmental signals? Is ligand binding conserved in these pathways?

## Highlights

All organisms possess biological timing methods, such as circadian rhythms and developmental timers, for coordination of life processes.

Mammals and *Drosophila* utilize functionally similar modes of genetic regulation to generate ~24-h circadian rhythms; however, key mechanisms and components are different.

Recent work demonstrates that *Caenorhabditis elegans* have conserved circadian regulators that not only drive *C. elegans* ~24-h cycles in adulthood but also transition from a developmental timer with ~8–10-h periods, offering insights into how genes can adapt to regulate distinct temporal programs.

Most chronobiological studies using *C. elegans* as a model organism have focused on genetic approaches, though recent work discussed in this review has integrated biochemical approaches with knowledge of mammalian and *Drosophila* circadian clocks.

## Glossary

Circadian rhythms: confer adaptive advantages by enabling organisms to predict, rather than respond to, changes in their environment, and are utilized by animals, plants, fungi, and some bacteria [100]. They are defined by three phenomena: (i) rhythms persist in constant conditions and have a period of ~24-h; (ii) they are entrainable with the ability to respond to external cues to synchronize with their environment; and (iii) they are temperature compensated, meaning that the ~24-h period is retained across biologically relevant temperatures [101].
Heterochronic pathways: developmental programs that require a specific sequence of events. In *Caenorhabditis elegans*, a conserved gene regulatory network coordinates cell fate programs to ensure they occur during the proper larval stage [62].
Molting: a developmental process seen in arthropods and nematodes, often controlled by hormonal signals. In *Caenorhabditis elegans*, this involves progressing through four larval stages by shedding its old cuticle (an outer exoskeleton-like structure) and synthesizing a new one to accommodate growth, a process essential for reaching adulthood [64].
Nuclear hormone receptors (NHRs): ligand-regulated transcription factors found exclusively in metazoans that regulate gene expression in response to steroid hormones.
Orphan receptors: nuclear hormone receptors that lack a known endogenous ligand.
Phosphoswitch: a model in which two phosphorylation sites on a protein determine how quickly it is degraded.
Transcription–translation feedback loops (TTFLs): generate oscillations in gene expression with a period of about a day for circadian rhythms. Transcription factors form the positive arm, inducing the expression of genes that make up the negative arm. After a delay, repressor proteins from the negative arm feedback to interact with or modify the positive arm, repressing transcriptional activity.
